# Leptospirosis as Cause of Febrile Icteric Illness, Burkina Faso

**DOI:** 10.3201/eid2408.170436

**Published:** 2018-08

**Authors:** Sylvie Zida, Dramane Kania, Albert Sotto, Michel Brun, Mathieu Picardeau, Joany Castéra, Karine Bolloré, Thérèse Kagoné, Jacques Traoré, Aline Ouoba, Pierre Dujols, Philippe Van de Perre, Nicolas Méda, Edouard Tuaillon

**Affiliations:** Centre Muraz, Bobo Dioulasso, Burkina Faso (S. Zida, D. Kania, T. Kagoné, J. Traoré, A. Ouoba, N. Méda);; University of Montpellier, Montpellier, France (S. Zida, A. Sotto, M. Brun, J. Castéra, K. Bolloré, P. Dujols, P. Van de Perre, E. Tuaillon);; Centre Hospitalier Universitaire de Nîmes, Nîmes, France (A. Sotto);; Centre Hospitalier Universitaire de Montpellier, Montpellier (M. Brun, P. Dujols, P. Van de Perre, E. Tuaillon);; Institut Pasteur, Paris, France (M. Picardeau)

**Keywords:** leptospirosis, febrile illness, jaundice, icteric, ELISA, MAT, PCR, bacteria, Burkina Faso, West Africa

## Abstract

Patients in Burkina Faso who sought medical attention for febrile jaundice were tested for leptospirosis. We confirmed leptospirosis in 27 (3.46%) of 781 patients: 23 (2.94%) tested positive using serologic assays and 4 (0.51%) using LipL32 PCR. We further presumed leptospirosis in 16 (2.82%) IgM-positive specimens.

Worldwide, approximately 1 million cases of human leptospirosis occur each year, resulting in ≈60,000 deaths (*1*). Although epidemiologic data for Africa are scarce, especially in semiarid and arid regions, some observations suggest that *Leptospira* spp. may be more prevalent than previously thought (*2*). In our study, we tested the hypothesis that leptospirosis is a cause of febrile jaundice in Burkina Faso.

## The Study

We conducted the study at Centre Muraz (Bobo Dioulasso, Burkina Faso), a central reference laboratory responsible for the national surveillance of yellow fever. We identified confirmed leptospirosis cases in accordance with World Health Organization criteria (*3*) by symptoms consistent with leptospirosis and a single microscopic agglutination test (MAT) titer >1:400, by detection of *Leptospira* DNA by PCR, or both. We identified presumptive cases by symptoms consistent with leptospirosis and the presence of IgM. Specimens testing negative for serologic and PCR were considered negative. We retrospectively tested samples collected during January 2014–July 2015 from adults and children with jaundice and fever >38.5°C for the presence of IgM against *Leptospira* spp. using an in-house ELISA (Technical Appendix). We assessed serum that tested positive by ELISA for antibodies to *Leptospira* bacteria in the bacteriology laboratory of Montpellier University Hospital (Montpellier, France), using MAT to confirm the serologic results with a panel of 7 reference serogroups. We also tested for leptospirosis specimens for which a sufficient volume of serum was available by MAT in the French National Reference Center (Paris, France), using a larger panel of 24 serogroups, including the first 7 serogroups (Table 1). We performed real-time PCR for leptospirosis at Centre Muraz using PCR (PUMA LEPTO Kit; Omunis, Clapiers, France) targeting the *lipL32* gene, which is present exclusively in pathogenic *Leptospira* spp. bacteria (*4*). 

**Table 1:** *Leptospira* spp. serogroups used for microscopic agglutination test*

Sample no.	Species	Serogroup	Serovar	Strain
1	*L. interrogans*	Australis*	Australis	Ballico
2	*L. interrogans*	Autumnalis	Autumnalis	Akiyami A
3	*L. interrogans*	Bataviae	Bataviae	Van Tienen
4	*L. interrogans*	Canicola*	Canicola	Hond Utrecht IV
5	*L. borgpetersenii*	Ballum*	Castellonis	Castellon 3
6	*L. kirschneri*	Cynopteri	Cynopteri	3522 C
7	*L. kirschneri*	Grippotyphosa*	Grippotyphosa	Moskva V
8	*L. interrogans*	Sejroe	Hardjobovis	Sponselee
9	*L. interrogans*	Hebdomadis	Hebdomadis	Hebdomadis
10	*L. interrogans*	Icterohaemorrhagiae	Copenhageni	Wijnberg
11	*L. noguchii*	Panama	Panama	CZ 214 K
12	*L. biflexa*	Semaranga	Patoc	Patoc 1
13	*L. interrogans*	Pomona*	Pomona	Pomona
14	*L. interrogans*	Pyrogenes	Pyrogenes	Salinem
15	*L. borgpetersenii*	Sejroë*	Sejroë	M 84
16	*L. borgpetersenii*	Tarassovi	Tarassovi	Mitis Johnson
17	*L. interrogans*	Icterohaemorrhagiae*	Icterohaemorrhagiae	Verdun
18	*L. weilii*	Celledoni	ND	2011/01963
19	*L. interrogans*	Djasiman	Djasiman	Djasiman
20	*L. borgpetersenii*	Mini	ND	2008/01925
21	*L. weilii*	Sarmin	Sarmin	Sarmin
22	*L. santarosai*	Shermani	Shermani	1342 K
23	*L. borgpetersenii*	Javanica	Javanica	Poi
24	*L. noguchii*	Louisiana	Louisiana	LUC1945

*Serogroups were tested in the Montpellier CHU laboratory and National Reference Center at Institut Pasteur (Paris, France). ND, not determined.

**Figure 1:**
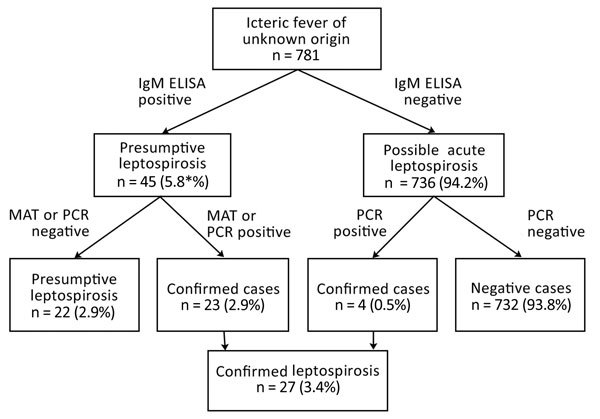
Flowchart used in study of leptospirosis in persons who sought medical attention for febrile jaundice, Burkina Faso. MAT, microscopic agglutination.

Of 781 samples, 45 (5.57%) tested positive for leptospira IgM by ELISA (Figure 1). Among those samples, 23 (2.94%) were positive by MAT (>1:400); consequently, these cases were considered to be confirmed. We considered 6 samples tested negative by MAT and 16 samples with MAT titer ranging from 1:100 to 1:200 (combined, 2.82%) to be presumptive cases. MAT results suggested the existence of multiple serogroups (Table 2), including reacting serogroups Australis, Ballum, Canicola, Grippotyphosa, Icterohaemorrhagiae, Pomona, and Sejroe. In addition, we performed MAT in the Leptospirosis National Reference Laboratory using a larger panel of serogroups applied to 33 ELISA-positive samples. Ten samples tested positive, and we confirmed the presence of all except the Ballum serogroup (data not shown). In 1 sample, we were able to identify Mini as an additional serogroup with a 1:400 titer. In addition to the serologic test, we confirmed leptospirosis cases by *lipL32* PCR in 4/781 (0.51%) samples. All were negative for IgM, but 3 had optical density just above the positive threshold; signal to mean value of the negative controls was between 2 and 3 (data not shown). Hence, screening by serologic assay plus PCR identified a total of 27 (3.46%) cases of confirmed leptospirosis. 

**Table 2:** Confirmed leptospirosis cases by serogroup using microscopic agglutination test, Burkina Faso*

Serogroup	No. positive, N = 23	Titer
Ballum	11 (1†)	1:400–1:1,600
Grippotyphosa	4 (1‡)	1:400–1:1,600
Australis	2	1:400 and,1:800
Canicola	1 1‡	1:400 and 1:800
Sejroe	1†(1§)	1:400
Icterohaemorrhagiae	1	1:800
Pomona	1	1:400
Mini	1§	1:400

*Values in parentheses represent duplicates, which are not included in the total count. †Titers were equal for serogroups Ballum and Sejroe. ‡Titers were equal for serogroups Grippotyphosa and Canicola. §Titers were equal for serogroups Mini and Sejroe.

**Figure 2:**
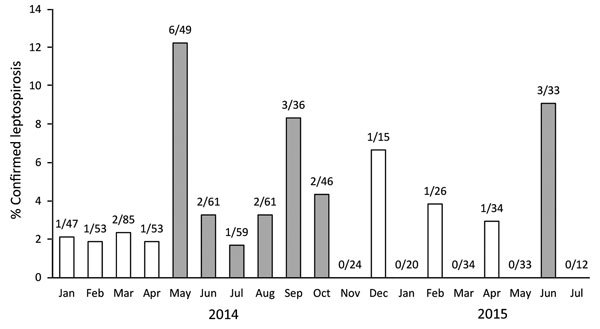
Confirmed cases of leptospirosis among samples received in Centre Muraz within the national network for yellow fever surveillance in Burkina Faso, January 2014–July 2015. White bars indicate months of the dry season, gray bars months of the rainy season. Numbers above bars indicate number of confirmed leptospirosis and the number of specimens tested.

Median age for all patients was 20 years (interquartile range [IQR] 12–30 years); 61% were male (p = 0.65 by χ^2^ test). We observed the highest number of confirmed cases in the age group 10–19 years (data not shown), but the frequencies were not significantly different when cases were analyzed by age group (p = 0.41 by χ^2^ test). This observation was not unexpected because the population of Burkina Faso is young; almost two thirds of the population is <25 years of age. There was no particular gender distribution for persons with confirmed cases (13 women and 14 men; p = 0.33 by χ^2^ test). The repartition of confirmed, presumptive, and negative cases according to rainy season (May–mid-October) versus dry season from mid-October–April was unequal (p = 0.0035 by χ^2^ test), with a trend for a higher proportion of confirmed cases among samples received during the rainy season when compared with negative cases (p = 0.065 by χ^2^ test; Figure 2).

Our data were in line with a recent publication estimating that some of the West Africa countries, including those in semiarid regions, may have among the highest rates of disability-adjusted life years due to leptospirosis; in Burkina Faso, the rate may be 60–70/100,000 population/year (*5*). Leptospirosis infections have been reported in various parts of West Africa in humans (*6*–*10*). Studies in Senegal and Mali have shown that cattle, pigs, and sheep are frequently infected (*11*,*12*). Detection of leptospirosis was also recently reported in rodents in Niamey, Niger, especially in urban agricultural settings (*13*). In Burkina Faso, agricultural and livestock sectors represent 30% of the gross domestic product and are the backbone of the economy with ≈80% of the working-age population involved in these activities (*14*). Hence, human exposure to *Leptospira* spp. bacteria is probably frequent. Studies conducted in Ghana on patients with febrile illness without an obvious cause of disease found a frequency of 3.2% of confirmed leptospirosis cases and 7.8% of probable cases among icteric patients (*2*). In our study, half of the probable leptospirosis cases characterized by clinical signs consistent with leptospirosis and screened positive for *Leptospira* IgM were confirmed by MAT with a titer >1:400; two thirds had a titer >1:100 that may also be leptospirosis cases. Collecting and testing a convalescent serum sample might have confirmed the presumptive cases. In addition, the MAT has been shown to be less sensitive than IgM detection using ELISA, especially in acute-phase specimens (*15*). The low rate of molecular test positivity may be explained by the rapid disappearance of *Leptospira* spp. bacteria in the blood at the time the antibody response becomes detectable. Furthermore, transportation from the field to the centralized laboratory and storage at −20°C for >1 year before testing had a potentially adverse effect on the detection of low levels of *Leptospira* DNA. 

Because our study was retrospective and based on single sample testing, we probably overlooked some cases of leptospirosis. The lack of detail about clinical symptoms and evolution was a limitation of this study. We recruited participants with the presence of jaundice in addition to fever; hence, it is probable that among the cases of confirmed leptospirosis, severe forms were overrepresented.

MAT provided a general insight into existing *Leptospira* serogroups within Burkina Faso, suggesting multiple reservoirs. However, cautious interpretation is invited because of the high degree of cross-reactions among different serogroups, especially in acute-phase serum samples. In addition, 2 instances of seroreactivity against Ballum serogroup observed in the first MAT performed in the Montpellier University Hospital were not confirmed by using an enlarged panel in the National Center for Leptospirosis. This finding may be related to prolonged sample storage and multiple freeze/thaw cycles before testing in the national reference laboratory.

## Conclusions

Leptospirosis appears to be an important cause of febrile jaundice in Burkina Faso, suggesting that leptospirosis is probably endemic in this country. Further studies are required to explore animal reservoirs and occupational risk factors associated with human leptospirosis. Awareness of leptospirosis among clinicians, funding for further study, and the possibility of conducting laboratory tests in the field are needed to clarify the extent of the problem in sub-Saharan Africa.

Technical Appendix Testing of serum samples for *Leptospira* spp. IgM by an in-house ELISA.
