# Supplementary material for: Leptospirosis as Cause of Febrile Icteric Illness, Burkina Faso
Source: Emerg Infect Dis. 2018 Aug;24(8):1569–72. doi: 10.3201/eid2408.170436 (PMC6056135; doi:10.3201/eid2408.170436)
Supplement: Technical Appendix — Testing of serum samples for Leptospira spp. IgM by an in-house ELISA. [file 17-0436-Techapp-s1.pdf]

Article DOI: https://doi.org/10.3201/eid2408.170436

Leptospirosis as Cause of Febrile Icteric Illness, Burkina Faso

Technical Appendix

IgM ELISA

We tested serum samples for the presence of IgM antibodies directed against *Leptospira* spp. by an in-house ELISA performed in Centre Muraz. The assay used an antigenic preparation of *Leptospira interrogans* serovar icterohaemorrhagiae provided by the French National Reference Centre for Leptospirosis at Pasteur Institute. Briefly, we inactivated cultures of bacteria at 56°C during 30 minutes, centrifuged them, and washed them in NaN_3 alkaline buffer. We centrifuged bacteria at 30 minutes at 4000 rpm and washed them with phosphate-buffered saline, and resuspended the pellets in NaHCO3 alkaline buffer. We coated polystyrene microtiter plates (Falcon Dominique Dutscher, Issy-les-Moulineaux, France) overnight at 4°C with 200 µL of *Leptospira* spp. suspension. On the second day, we rinsed the plates twice with $NaHCO_3$ alkaline buffer and repeated the coating step once in the same conditions. After washing, we packaged the plates and stored them at –20°C until use. The antigen-covered plates were stable for 3 months. We used a phosphate buffered saline (PBS)/Tween buffer (manufactured in-house) containing milk to make 1:100-fold dilution of the serum samples. We dispensed 200 µL per well, incubated them for 1 hour at 37°C, and washed them 3 times in NaCl/Tween buffer (manufactured in-house). We added alkaline phosphatase conjugated goat anti–human IgM (Rockland TEBU BIO, Rockland Inc., Limerick, PA, USA) at a dilution of 1:1,600 in PBS/Tween and incubated for 1 hour at 37°C. After washing 3 times, we added 200 µL of substrate (4 nitrophenyl phosphate [Reference A12310.06] Alfa Aesar, A Johnson Matthey Company, Heysham, UK) for 15 min at room temperature. We measured optical density at 405 and 630 nm. We used 5 negative controls and 2 positive controls for each microtiter plate. We calculated the positive threshold based on the value of negative controls (mean value × 3). Hence, samples with ratio of signal to mean value of the negative controls >3 were considered positive.
